# Harnessing bioactive compounds from *Cannabis sativa* residue to improve rumen fermentation and reduce methane production: in silico, in vitro, and in situ nylon bag studies

**DOI:** 10.1186/s12917-025-04985-5

**Published:** 2025-10-14

**Authors:** Patipan Hnokaew, Chaiwat Arjin, Apinya Satsook, Joshua Nizel Halder, Suriya Tateing, Nuttha Potapohn, Korawan Sringarm

**Affiliations:** 1 Faculty of Agriculture, Department of Animal and Aquatic Sciences, Chiang Mai University, Chiang Mai, 50200 Thailand; 2 Office of Research Administration, Chiang Mai University, Chiang Mai, 50200 Thailand; 3 Faculty of Agriculture, Department of Plant and Soil Sciences, Chiang Mai University, Chiang Mai, 50200 Thailand

**Keywords:** Methyl-coenzyme M reductase, *Cannabis sativa* L. residue, Cannabinoids, Methane emissions, Ruminal fermentation

## Abstract

The inhibition of methyl-coenzyme M reductase (MCR) effectively suppresses ruminal methanogen activity, thereby mitigating enteric methane emissions in ruminants. However, the development of highly specific and environmentally sustainable inhibitors remains a significant challenge. This study aimed to identify key bioactive compounds from *Cannabis sativa* L. residue (CSR) that function as novel methane inhibitors that specifically target MCR by computational molecular screening. Cannabidiol (CBD) and delta-9-tetrahydrocannabinol (THC) were identified as potential candidate compounds using the molecular docking technique. CBD and THC were found to be compounds that can navigate through a narrow channel and bind to the active sites of MCR, with calculated binding free energies of -8.8 and -5.5 kcal/mol, respectively. Furthermore, the effects of CSR supplementation levels on rumen fermentation, nutrient digestibility, methane production, and the ruminal microbial population were investigated through in vitro rumen fermentation simulations, as well as in situ digestibility analysis according to a completed randomized design (CRD). Compared with the control, supplementation with 2% total DM substrate in a total mixed ration (TMR) diet resulted in a 34% decrease in methane production. In addition, CSR increased the molar proportion of propionic acid and the concentration of ammonia-nitrogen (NH_3_-N), whereas the molar proportion of acetic acid and the acetic acid to propionic acid ratio decreased. At the bacterial level, the population of *R. flavefaciens* decreased with CSR supplementation. At the archaeal level, the population of *Methanobacteriales* decreased with CSR supplementation. These findings suggest that CSR has the potential to be used as a novel natural additive for inhibiting ruminal methane production. Further in vivo studies are recommended to validate these findings.

**Supplementary Information:**

The online version contains supplementary material available at 10.1186/s12917-025-04985-5.

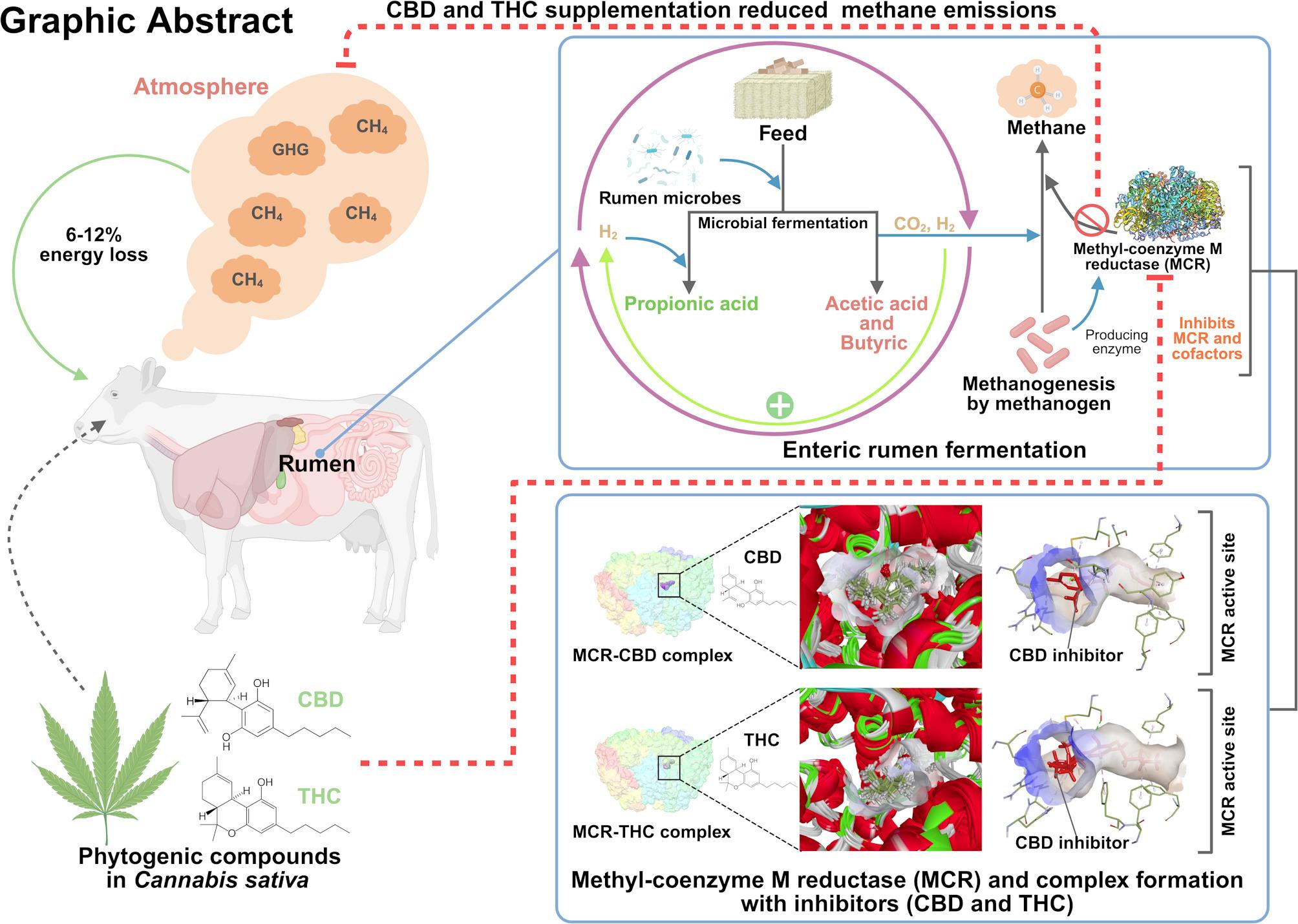

## Introduction

**Fig. 1:**
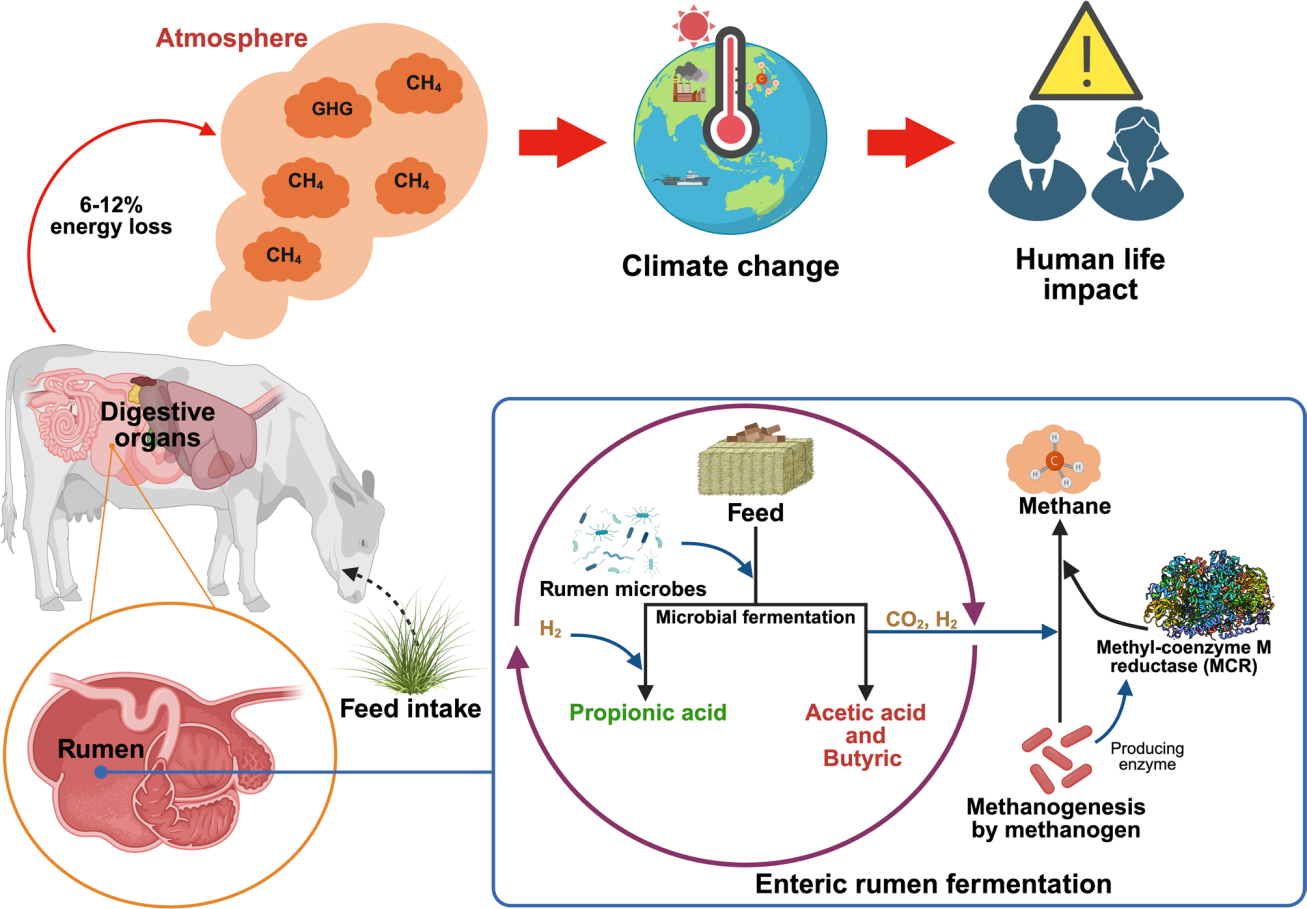
Enteric methane formation processes in the rumen Adapted from Bature et al. [9]; Sadid et al. [10]; Dinakarkumar et al. [11]; Place et al. [12]

In recent years, there has been a heightened focus on the environmental implications of meat and dairy production, particularly regarding methane (CH_4_) emissions and their significant contribution to the exacerbation of global warming [1]. Methane from livestock production is a significant source of greenhouse gases, contributing to climate change [2]. Furthermore, methane emissions represent a substantial energy loss in ruminant animals, and are produced by methanogenic archaea through methanogenesis. This process requires the enzyme methyl-coenzyme M reductase (MCR) to convert carbon dioxide and hydrogen into methane [2] (Fig. 1). It is estimated that up to 44% of greenhouse gas emissions are attributed to this sector [3], with approximately 40% of emissions being byproducts from feed fermentation in the rumen [4]. Consequently, there is significant interest among governments and scientists in developing strategies aimed at mitigating methane emissions originating from ruminants. Various strategies for reducing enteric methane emissions have been proposed and examined in recent decades. Managing animals and feed, diet formulation, rumen manipulation, and feed additives have been found to be practical and effective approaches to reduce enteric methane emissions [5–8].

Several viable enteric methane inhibitors, including alternative electron receptors [13], halogenated methane analogs (HMAs) [14], and methyl-coenzyme M analogs [15], have been proposed in recent years. Chemical compounds that can bind to the active site of MCR are promising for inhibiting enteric methane emission. MCR plays a crucial role in the methanogenesis pathway by catalyzing the reduction of methyl coenzyme M with coenzyme B, resulting in the formation of methane and the heterodisulfide compounds of coenzyme M and coenzyme B [16]. Methane production can begin with various substrates, depending on the methanogen species, including CO₂, methanol, formate, acetate, and methylamine. However, all three methanogenesis pathways converge in the final two steps of the process. Since MCR plays a role in the last step, it serves as a shared target across all methanogen species, making it a key enzyme for strategies aimed at inhibiting methanogenesis in ruminants [17]. Previous studies have shown that various methyl-coenzyme M analogs, such as 2-bromoethanesulfonate (BES) [18], 2-chloroethanesulfonate (CES) [19], 3-bromopropanesulfonate (BPS) [19], and 3-NOP [20], can competitively and specifically inhibit MCR activity and thus decrease methane production. Although these synthetic compounds have been proven effective, they may exert a range of adverse effects on animal health, including damage to the nervous system, suppressed immunity, developmental and reproductive issues, as well as liver and kidney damage [21]. The observation that methanogens can adapt to BES raises concerns regarding the use of coenzyme M analogs. In addition, increasing their dose could lead to methanogen resistance [19]. Bromoform is widely recognized as an effective compound for the inhibition of methane production, and its negative impacts on animal health and the atmosphere environment have been extensively studied [22]. In contrast to synthetic chemical compounds with adverse effects, phytochemical products are generally considered natural alternatives that present a lower threat to the environment or less risk of triggering resistant microorganisms in the animal.

Previous studies have show that various phytonutrient compounds such as phenolic acids, tannins, flavonoids, anthocyanidins, carotenoids, and caffeine can be efficiently utilized to inhibit rumen methanogens and reduce enteric methane emissions in ruminants [3, 23]. Nevertheless, the primary mechanism by which these compounds mitigate methane is through their impact on ruminal protozoa or ruminal fermentation patterns, and their capacity to specifically inhibit methanogenesis is considerably limited. Identifying target molecules with specific inhibitory effects on methanogens from phytochemical compounds of plants is an enormous and challenging task. However, computer-aided drug discovery (CADD) techniques, also known as in silico methods, are becoming more appealing for finding methanogenesis inhibitors because of their scalability, time efficiency, and cost-effectiveness [24]. In silico docking is a computational approach used in drug discovery and molecular biology to predict the binding orientation and affinity of a small molecule, such as an inhibitor, within the active site of a protein [17]. These insights can aid in the development of new inhibitors and the enhancement of current ones. By docking a selected library of bioactive compounds of interest into a high-resolution crystal structure of the target enzyme, prospective candidates can be identified. The aforementioned information accordingly indicates that methane synthesis in the rumen depends on the activity of MCR, a key enzyme found in all methanogens [25], making MCR the best choice as a target receptor for in silico studies.

*Cannabis sativa L*. is an annual plant belonging to the Cannabaceae family [26]. In the past, *C. sativa* has been employed as a medicinal plant in a variety of cultures worldwide to treat a variety of diseases. A recent study has shown that the antioxidant capacity of finishing calves can be enhanced by *C. sativa* biomass [27]. Additional research has demonstrated that *C. sativa* has no adverse effects on carcass characteristics or productive performance in sheep [28] and goats [29]. Feeding beef cattle with hempseed byproducts containing cannabinoids, derived from the same plant species as *C. sativa*, under conditions comparable to commercial farming does not result in the accumulation of cannabinoids in edible tissues such as muscle, liver, and kidney [30]. Although residues of cannabinoids have been detected in the milk of cattle fed cannabinoid-rich industrial hemp silage, the transfer rate of cannabinoids from feed to milk is only approximately 0.2% [31]. However, appropriate inclusion levels and feeding management strategies may help mitigate cannabinoid residues in milk. Furthermore, *C. sativa* has been reported to possess powerful anti-inflammatory properties [32, 33], antioxidant [26], antimicrobial [34], and antifungal properties [35]. Nevertheless, the utilization of *C. sativa* declined in the early 20th century as a result of its classification as an illicit substance, which was attributed to its psychoactive component [36]. Following the legalization of cannabis in numerous countries, there has been a surge in demand for cannabis products for both recreational and medicinal purposes [37]. In 2019, Thailand was the first and only nation in Southeastern Asia to legalize cannabis for both medical and research purposes [38]. At the outset, eight government institutions were granted licenses by the Thai Food and Drug Administration to cultivate, produce, and process cannabis for medical-grade products [39]. The number of producers who subsequently met the required standards to obtain a government license grew quickly [40]. Typically, primary cannabis products are made from *C. sativa* inflorescences and roots [41, 42], and their leaves and stalks are referred to as cannabis residues (CSRs). Consequently, investigating the potential applications of these residues to accomplish zero-waste production in the *C. sativa* industry is imperative. This is particularly noteworthy, as prior research has demonstrated that cannabis residues contain a variety of medicinal compounds, such as delta-9-tetrahydrocannabinol (THC), cannabidiol (CBD), and other phytochemicals such as flavonoids and terpenes [32, 43]. Sopien et al. [42] reported that cannabis residues contain CBD (734.3 mg/kg) and THC (1005.9 mg/kg). However, the study of the mechanisms of action of the phytochemicals CBD and THC, found in cannabis, regarding their potential to inhibit the activity of MCR and reduce methane production in ruminants, represents a novel and promising research direction. Although scientific evidence in this area remains limited, these compounds may offer a potential solution to mitigate the environmental impacts of ruminant livestock production. Therefore, the objective of this study was to identify major bioactive compounds from CSR that may serve as novel methane inhibitors that specifically target MCR by molecular docking technology and molecular dynamics technology and then verify their rumen methane mitigation capacity through an in vitro rumen fermentation technique, as well as in situ technique to study its impact on digestibility in the rumen.

## Materials and methods

### In Silico approaches

Two structural compounds, CBD (CID: 644019) and THC (CID: 16078), were obtained from the PubChem database [44]. Their three-dimensional structures were optimized using the steepest descent algorithm within the MMFF94 force field through the Avogadro program [45] before being processed for ligand preparation in AutoDockTools software under MGLTools (version 1.5.7). The crystal structure of methyl-coenzyme M reductase (MCR, PDB ID: 5A8K) was retrieved from the RCSB protein databank (https://www.rcsb.org/). The MCR protein was carefully checked and cleaned for the missing residues before being subjected to MGLTools software for protein preparation. Molecular docking was performed to predict the ligand’s binding pose within the protein receptor and analyze protein-ligand interactions. This was conducted using AutoDock Vina software [46] (The Scripps Research Institute, La Jolla, CA, USA) on the Linux platform, with binding energies calculated and reported in kcal/mol for ligand-protein interactions. The key residues used to define the grid box were determined on the basis of previous studies [11, 15]. The grid box was designed to target the active site, with dimensions of (x = 22, y = 20, z = 24) and a center at (x = 49.85, y = 16.12, z = 25.80). A grid spacing of 0.375 Å was applied to encompass the key binding site residues of the 5A8K protein, which was then adopted for simulations. UCSF Chimera, Biovia Discovery Studio Visualizer, and Ligplus tools were used to study protein-ligand interactions after all the ligand molecules were docked to the active site of the 5A8K protein. The highest-ranked ligands, which exhibited the most negative and favorable interactions, were selected for further molecular dynamics (MD) simulation analysis. MD simulation were used to evaluate the conformational changes and structural stability of the ligand-protein complex throughout the binding process. Classic molecular dynamics (MD) simulations were conducted using GROMACS 2022 [47, 48]. In summary, the topology and parameters for the protein were generated using the AMBER99SB force field [49], whereas those for the ligand were obtained from the ATB server [50]. The complexes were solvated by adding water molecules modeled with the SPC framework, and counterions such as sodium (Na⁺) and chloride (Cl⁻) were introduced to neutralize the system. Energy minimization was performed using the steepest descent algorithm with a tolerance of 1000 kJ/mol/nm, followed by 1000 ps of equilibration in both the NVT and NPT ensembles, with position restraints applied to the ligand during the equilibration step. The simulation parameters were set at a temperature of 300 K and a pressure of 1 bar for each system. A 2 fs integration step was applied. The production phase lasted for 600 ns, with snapshots recorded every 20 ps for subsequent analysis. Upon completing the simulation, the root-mean-square deviation (RMSD) and root-mean-square fluctuation (RMSF) were computed using the utilities available in the GROMACS package.

### Animal ethics

All procedures involving the use and management of animals were officially assessed and approved by the Institutional Animal Care and Use Committee (Agricultural Animals), which approved all the experimental protocols used in this study (record no. AG01005/2568). The animals involved in this study were owned and managed by the Department of Animal and Aquatic Sciences, Faculty of Agriculture, Chiang Mai University, Chiang Mai, Thailand. Additionally, we ensured that all methodologies adhered to established regulatory and ethical frameworks, consistent with the principles outlined in the ARRIVE guidelines.

### Cannabis residue collection

*Cannabis sativa* L. residues (CSRs) were collected in September 2024 from Atlanta Medicare Co., Ltd, Bangkok, Thailand. The fresh CSR was dehydrated in an oven tray dryer at 60 °C for 48 h until completely dry. After drying, the CSR was pulverized using a hammer mill (Retsch SM100) and passed through a 2-mm mesh screen to acquire the CSR powder, which was subsequently stored at room temperature in airtight bags for future use.

### Experimental design, dietary treatments, and chemical analysis

This study employed the in vitro gas production method and the in situ nylon bag technique at different incubation periods. The dietary treatments included a total mixed ration (60:40 roughage-to-concentrate ratio) with the addition of *C. sativa* L. residue (CSR) at 0% (T1), 0.5% (T2), 1% (T3), and 2% (T4) total DM substrate. All dietary samples were dried in an oven at 60 °C and ground to pass through a 1-mm filter (Retsch SM100). Each experimental dietary sample and CSR powder were evaluated for dry matter (DM), organic matter (OM), and ether extract following AOAC methods [51]. The nitrogen content was measured via a Nitrogen Analyzer (Leco FP828, LECO Corporation, Saint Joseph, MI, USA) to assess the crude protein level. The fiber contents, including neutral detergent fiber (NDF) and acid detergent fiber (ADF), were analyzed following the method established by Van Soest et al. [52]. The compositions and feed formulations of the experimental diets are shown in Table 1. The method outlined by Muangsanguan et al. [54]. was subsequently used to assess the phytonutrient contents and antioxidant activity of the CSR powder. The phytonutrient content includes the total phenolic content (TPC). The analysis of cannabidiolic acid (CBDA), cannabinol (CBN), cannabidiol (CBD), tetrahydrocannabinolic acid (THCA), and delta-9-tetrahydrocannabinol (THC) was carried out using HPLC/UV (Shimadzu HPLC LC-20 A, Japan) according to the method outlined by Sopian et al. [42]. The evaluation of antioxidant capacities was performed using three different techniques: the 2,2-diphenyl-1-picrylhydrazyl (DPPH) radical scavenging method, the 2,2-azino-bis(3-ethylbenzthiazoline-6-sulphonic acid) (ABTS) radical scavenging activity, and the ferric reducing ability power (FRAP) method.

**Table 1:** The formulation and nutritional composition of the experimental diets

Item	Treatment^1^			
	T1	T2	T3	T4
Ingredients (g/kg of dry matter)				
Napier grass (45 day cutting age)	200	200	200	200
Rice straw	400	400	400	400
Rice Bran	100	100	100	100
Soybean meal	120	120	120	120
Ground corn	100	100	100	100
Soy Sauce Meal	50	50	50	50
Urea	10	10	10	10
Dicalcium phosphate	10	10	10	10
Mineral mixed^2^	10	10	10	10
CSR powder	0	5	10	20
Chemical composition				
Dry matter (DM), g/kg	906	907	908	907
Organic matter, g/kg DM	876	865	865	864
Crude protein, g/kg DM	178	176	177	175
Ether extract, g/kg DM	44	42	45	43
Neutral detergent fiber, g/kg DM	627	629	626	632
Acid detergent fiber, g/kg DM	382	388	384	387
Gross energy, Kcal/kg	3547	3626	3616	3600
Bioactive compounds^3^				
Total phenolic, mM GAE/g DM	3214.35	3463.68	3552.27	3600.53
CBDA, mg/kg DM	nd	3.42	4.98	7.99
CBD, mg/kg DM	nd	43.82	54.92	90.20
CBN, mg/kg DM	nd	2.43	2.83	3.81
Total CBD, mg/kg DM	nd	46.82	59.29	97.21
THCA, mg/kg DM	nd	6.09	6.24	6.34
THC, mg/kg DM	nd	0.52	1.10	2.30
Total THC, mg/kg DM	nd	5.86	6.57	7.86
Antioxidant activities				
DPPH, IC50 (mg/mL)	16.95	16.11	14.62	13.15
ABTS, IC50 (mg/mL)	61.89	57.71	47.71	28.93
FRAP, mM Fe^2+^/g DM of sample	229.35	252.01	339.50	449.60

^1^ Treatment: T1; 0% CSR, T2; 0.5% CSR, T3; 1.0% CSR, T4; 2.0% CSR. ^2^ Contains per kg premix: vitamin A 10,000,000 IU; vitamin D 1,600,000 IU; vitamin E 70,000 IU; Fe 50 g; Mn 40 g; Zn 40 g; Cu 10 g; I 0.5 g; Se 0.1 g; Co 0.1 g. ^3^*GAE* gallic acid equivalent, *CBDA* cannabidiolic acid, *CBD* cannabidiol, *CBN* cannabinol, *THCA*; tetrahydrocannabinolic acid, THC; delta-9-tetrahydrocannabinol, Total CBD was calculated based upon CBD + 0.877 * CBDA (Stevens et al.) [53], Total THC was calculated based upon THC + 0.877 * THCA (Stevens et al.) [53]. IC_50_; the 50% half maximal inhibitory concentration (mg/mL), mM Fe^2+^/g DM pf sample; µM ferrous ion per DM pf sample. nd; not detected

### In vitro rumen substrate and incubation

The animals were used and carried out at the farm of the Department of Animal and Aquatic Sciences, Faculty of Agriculture, Chiang Mai University, Chiang Mai, Thailand. Three male rumen-fistulated Thai crossbred beef cattle (Brahman x Thai Indigenous) with body weights (BWs) of 450 ± 20.0 kg were used in this study. The trial donor cattle received a concentrate diet at 1% of BW daily in addition to ad libitum access to Napier grass. The concentrate diet contained 160 g/kg crude protein, which was fed twice a day at 7:00 AM and 5:00 PM. Fresh rumen fluid was collected before the morning feeding. The rumen fluid from each donor was filtered through four layers of cheesecloth and mixed in equal volumes. The mixed rumen fluid was then transferred into a 1,500 mL sterile bottle maintained at 39 °C with CO_2_. The sample was promptly transported to the laboratory within 20 min and continuously infused with CO_2_ until use. The preparation of buffer solutions and minerals was carried out according to the method described by Menke et al. [55]. The ruminal buffer mixture was blended with rumen fluid at a 1:2 ratio (mL/mL) and stirred at 39 °C under CO_2_-containing conditions. For each dietary treatment, the substrate (0.5 g) was weighed and combined with 40 mL of the ruminal fluid mixture into 120 mL serum bottles. The bottles were then sealed with aluminum caps and rubber stoppers and incubated at 39 °C.

### In vitro rumen fermentation profile and nutrient degradability

In the in vitro study, the experimental procedure was carried out in three independent runs, each conducted at 7-day intervals to minimize inter-run variability and to ensure the robustness and reproducibility of the results. During each run, all treatment groups were incubated simultaneously under standardized and controlled conditions to maintain consistency across replications. The specific procedures within each run were as follows: gas production kinetics were evaluated using three serum bottles for each treatment, along with three blank serum bottles for comparison. Gas production was measured using a glass syringe injection at sample bottles to determine the gas volume at each time point, with recordings taken immediately after incubation at the following time points: 0, 2, 4, 6, 8, 10, 12, 24, 48, 72, and 96 h. The gas production data were analyzed via the Ørskov and McDonald [56] equation as follows:

$$\mathrm Y\;=\;\mathrm a\;+\;\mathrm b\;(1\;-\;\mathrm e^{-\mathrm{ct}})$$

where Y represents the gas production at time t (mL), a represents the gas production from the soluble fraction (mL), b represents the gas produced from the insoluble fraction (mL), c represents the gas production rate constant for the insoluble fraction (mL/h), and t represents the incubation time (h).

After incubation for 12 and 24 h, headspace samples from the serum bottles (3 bottles per treatment for each incubation time) were collected to determine the methane concentration in the vacuum tube, which was subsequently analyzed via gas chromatography (CheckMate 9900, PBI Dansensor, Denmark) with helium as the carrier gas at a flow rate of 30 mL/min. A DB-WAX UI type column (HayeSep Q 6FT × 1/8IN × 2.1MM) was controlled at 300 °C and connected to a thermal conductivity detector (TCD). Next, the ruminal liquor incubated samples (3 bottles per treatment) were collected to analyze the rumen pH, ammonia-nitrogen (NH_3_-N) content, volatile fatty acid (VFA) content, and microbial population. The ruminal pH parameters were immediately measured with a portable digital pH meter (Mettler Toledo, Switzerland). The ruminal liquor incubated samples were divided into two parts. The first portion (20 mL) was mixed with 5 mL of 1 M H_2_SO_4_ and stored at − 20 °C for NH_3_–N analysis, which was conducted using a spectrophotometer (UV/VIS spectrophotometer, PG Instruments Ltd., London, United Kingdom) according to the methods of Chaney and Marbach [57], whereas the VFA concentrations were measured via gas chromatography (GC) with a Shimadzu GC-2030 instrument (Kyoto, Japan), following the description of Filipek and Dvorak [58]. The second part (5 mL) was preserved via a DNA/RNA Shield (Zymo Research, USA) at a ratio of 1:1 (mL/mL) in a plastic bottle to prevent degradation during sample collection and was stored at − 80 °C for DNA extraction. For in vitro digestibility, degradability measurements (3 bottles per treatment for each incubation time) were assessed in terms of dry matter degradability (IVDMD) and organic matter degradability (IVOMD). After 12 and 24 h of incubation, the samples were collected and stored at −20 °C to halt microbial activity for subsequent analysis, in accordance with the procedure outlined by Tilley and Terry [59]. After the IVDMD value was determined at 105 °C, the same crucibles were heated at 550 °C for 4 h, weighed, and then used to assess the degradability of organic matter (OM).

### Relative quantification of specific ruminal microbes

Prior to DNA extraction, fermenter fluid samples were thawed at 4 °C and homogenized. Total genomic DNA was extracted from approximately 1.5 mL of homogenized fluid sample using the QIAamp PowerFecal Pro DNA Kit (QIAGEN, Valencia, CA, USA) via a modified bead-beating protocol. The concentration and purity of the extracted DNA were detected by NanoDrop2000 spectrophotometry (Thermo Scientific, Wilmington, DE, USA) and gel electrophoresis. Real-time polymerase chain reaction (RT-PCR) assays were performed using an RT-PCR machine (CFX96 Real-Time system, Bio-Rad, USA). The reaction was carried out using SYBR Green Supermix (QPK-201, Toyobo Co., Ltd., Tokyo, Japan) in a final volume of 20 µL, which included forward and reverse primers (10 pmol each), SYBR Green Supermix, and template DNA. All reactions were performed in triplicate. The primer sets used for the amplification of *Ruminococcus albus*,* Ruminococcus flavefaciens*,* Fibrobacter succinogenes*, *Megasphaera elsdenii*, and *Methanobacteriales* are shown in Supplementary Table 1. After real-time PCR, the relative population abundances of the specific bacteria were expressed as a ratio of total bacterial 16 S rDNA according to the following equation: relative quantification = 2^−(∆Ct target−∆Ct total bacteria)^, where Ct is the threshold cycle. A negative control without template DNA was used in every real-time PCR assay for each primer.

### In situ nylon bag measurement

The rumen fluid donor cattle were fed the basal diet (T1; ingredients and nutrient composition shown in Table 1) for an adaptation period of 21 days prior to the in situ experiment to ensure rumen stability. The animals utilized in the nylon bag technique study were maintained and managed at the farm of the Department of Animal and Aquatic Sciences, Faculty of Agriculture, Chiang Mai University, Chiang Mai, Thailand. The characteristics of the three male rumen-fistulated Thai crossbred beef cattle used in this experiment were the same as those described earlier. The in situ nylon bag approach was used to determine dry matter (DM) and organic matter (OM) according to Ørskov and McDonald [56]. Approximately 1 g of the feed sample was collected in an F57 fiber filter bags (Ankom Technology, Macedon, NY, USA) with a pore size of 25 μm. All the samples were prepared in triplicate and incubated in the rumen of each animal for 3, 6, 12, 24, 48, 72, or 96 h. Fiber filter bags were incubated for the indicated durations in the rumen and removed at the same time. After the specified incubation periods, the bags were removed from the rumen, washed immediately with cold tap water until clear, and then dried in a forced-air oven at 60 °C for 48 h. The control bags, which were not incubated (0 h), were washed and dried under the same conditions. DM and OM of the residual feeds were determined using the AOAC (2000) [51]. The percentages of DM and OM disappearance at each incubation time were calculated on the basis of the proportion remaining after incubation in the rumen via SigmaPlot version 15.0 software, following the methods outlined by Ørskov and McDonald [56] as follows:

$$\mathrm P\;=\;\mathrm a\;+\;\mathrm b\;(1\;-\;\mathrm e^{-\mathrm{ct}})$$

where P is the disappearance rate at time t (%), a is the intercept representing the portion of DM or OM solubilized at the initiation of incubation (time 0), b is the fraction of DM or CP potentially degradable in the rumen, c is the rate constant of disappearance of fraction b, and t is the time of incubation (h).

The effective degradability (ED) of dry matter (EDDM) and the effective degradability of organic matter (EDOM) were calculated using the following equation:

$$\mathrm{ED}\;=\;\mathrm a\;+\;\lbrack\mathrm{bc}/(\mathrm c\;+\;\mathrm k)\rbrack$$

where k is the estimated rate of outflow from the rumen (0.05 h^−1^), and a, b, and c are the same parameters as described earlier.

### Statistical analysis

Prior to statistical analysis, normality and homogeneity of variance were tested using the Shapiro-Wilk test and Levene’s test. The investigation results were statistically analyzed as a completed randomized design (CRD) statistical run using the aov function in the R statistical program (version 4.3.1) [60], as in the model below:

$${\mathrm Y}_{\mathrm{ij}}\;=\;\mathrm\mu\;+\;{\mathrm\tau}_{\mathrm i}\;+\;{\mathrm\varepsilon}_{\mathrm{ij}},$$

where Y_ij_ is the observation, µ is the overall mean, τ_i_ is the treatment effect (the CSR supplementation levels at 0, 0.5, 1, and 2% DM of total substrate), and ε_ij_ is residual error. The results are expressed as mean values along with the standard error of the mean (SEM). Differences between treatment means were assessed using Duncan’s new multiple range test, and a P-value of less than 0.05 was considered statistically significant. Orthogonal polynomial contrasts were conducted to assess whether the effect of CSR levels followed a linear, quadratic, or cubic pattern.

## Results

### Molecular Docking and molecular dynamics simulation studies

**Fig. 2:**
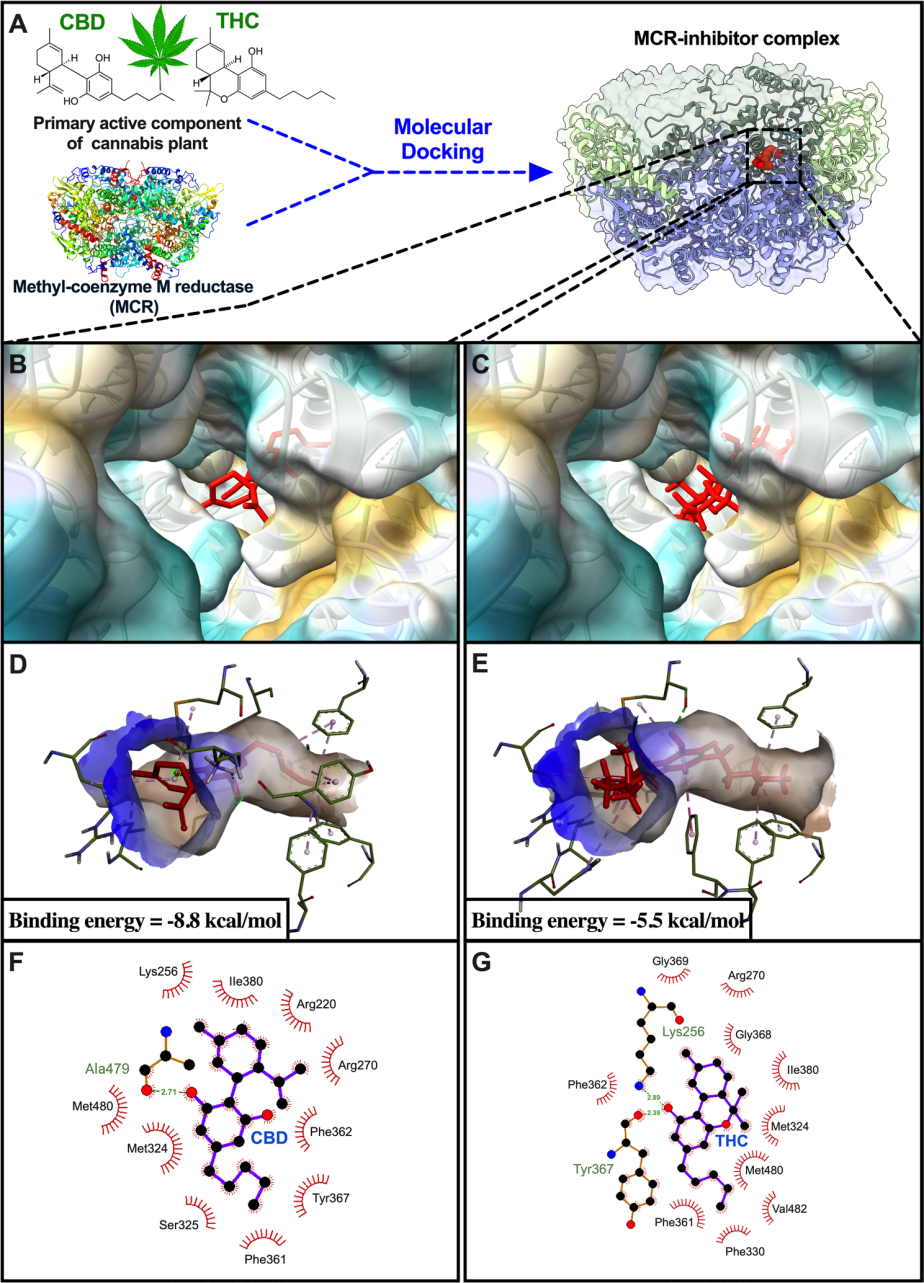
Structural overview of methyl-coenzyme M reductase (MCR) and its active site location. **(A)** Molecular docking flow chart, and docking surface pocket poses of **(B)** cannabidiol (CBD) and **(C)** delta-9-tetrahydrocannabinol (THC); 3-dimensional plots of protein − ligand interactions of **(D)** CBD and **(E)** THC in the active site of MCR; 2-dimensional plots of protein − ligand interactions of **(F)** CBD and **(G)** THC in the active site of MCR

**Fig. 3:**
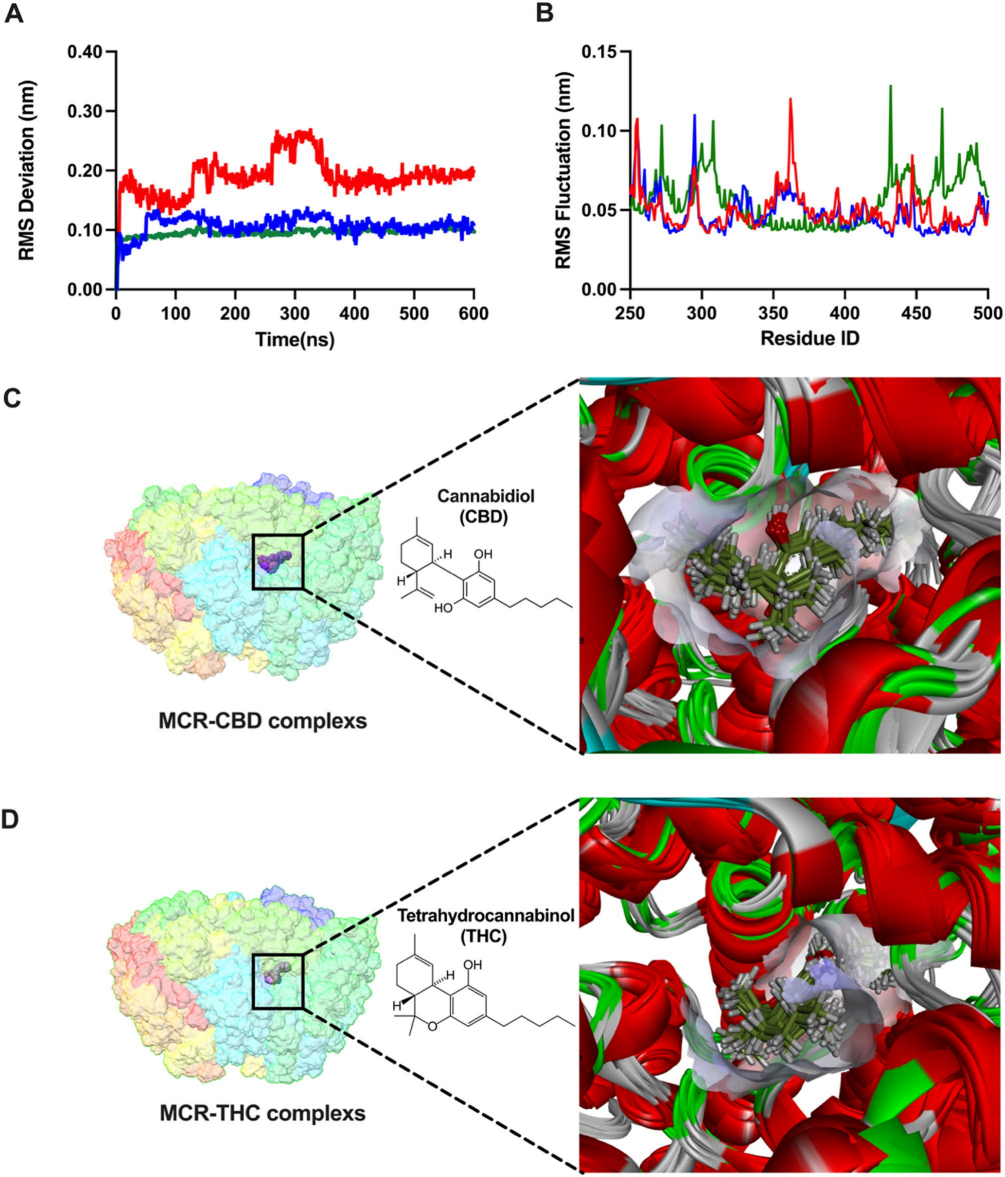
Illustration showing the results of the molecular dynamics simulations of the cannabidiol (CBD) and delta-9-tetrahydrocannabinol (THC) complexes. **(A)** Root-mean-square deviations (RMSDs) and **(B)** root-mean-square fluctuations (RMSFs) of the protein backbone of unbound and bound MCRs with two inhibitors: green represents unbound methyl-coenzyme M reductase (MCR), red represents MCR-CBD complex, and blue represents MCR-THC complex. Molecular overlay of MCR-inhibitor complexes: **(C)** CBD, **(D)** THC

Two natural compounds (CBD and THC), which are key components of CSR, were docked into the binding site of the MCR protein receptor, with binding energy values of − 8.8 and − 5.5 kcal/mol, respectively. On the basis of the binding affinity and ligand conformation data, CBD demonstrated a greater binding energy than did THC (Fig. 2A-E). CBD interacted with Lys256, Ile380, Arg225, Arg270, Phe362, Tyr367, Phe361, Ser325, Met324 and Met480 with hydrophobic interactions as well as with Ala479 with hydrogen bonding (Fig. 2F). THC interacted with Gly369, Arg270, Gly368, Ile380, Met324, Met480, Val482, Phe330, Phe361, and Phe362 via hydrophobic interactions as well as with Lys256 and Tyr367 with hydrogen bonding (Fig. 2G). To determine the stability of the MCR-inhibitor complex, we calculated the RMSD and RMSF during a 600-ns simulation. Figure 3A shows the RMSD plots of the protein backbone and ligand of the selected natural compounds. The RMSD of the backbone of the complex averaged between 0.1 and 0.3 nm, whereas the RMSD of the ligand varied between 0.1 and 0.2 nm. Both CBD and THC presented low RMSD values, suggesting minimal conformational changes and favorable initial poses. All protein‒ligand complexes achieved equilibrium within 50 ns of simulation and remained stable thereafter. The RMSF plot was used to assess the fluctuation positions, revealing that, for each scenario, the greatest fluctuation occurred at the binding sites (Fig. 3B). All the protein backbones exhibited slight fluctuations of less than 0.1 nm, reflecting a stable equilibrium state. Our calculations suggest that both CBD and THC could fit into the active site of MCR and remain stably bound throughout the simulation duration. To investigate the dynamics of MCR receptor binding with various inhibitors, snapshots (400–600 ns) of the MD trajectories were collected and categorized into protein‒ligand conformations with 20 ns intervals. The molecular overlay feature in Discovery Studio Visualizer was used to superimpose the molecules on the basis of the similarities in the protein structures. The molecular overlay results indicated that the structures of CBD (Fig. 3C) and THC (Fig. 3D) remained stable on the MCR receptor.

### Nutrient composition of the dietary treatments and CSR

The feed ingredients of the CSR supplementation treatments and their nutrient compositions are presented in Table 1. As CSR inclusion in the TMR diet increased, the bioactive compound levels (TPC, CBDA, CBD, CBN, total CBD, THCA, THC, and total THC) and antioxidant activities (DPPH, ABTS, and FRAP) correspondingly increased. The results of the nutrient composition, phytochemical content, and antioxidant activities of CSR are presented in Table 2. CSR is high in crude protein (295 g/kg DM). The CSR used in this study contained a total phenolic content of 4663.01 mM GAE/g DM. The antioxidant potential of CSR, as measured using the DPPH, ABTS, and FRAP methods, was 12.78 IC_50_ (mg/mL), 18.38 IC_50_ (mg/mL), and 1664.49 mM Fe²⁺/g DM of sample, respectively. In addition, the CSR was found to be rich in cannabinoids, with CBDA at 1560.88 mg/kg DM, CBD at 7829.28 mg/kg DM, CBN at 1574.34 mg/kg DM, total CBD at 9198.17 mg/kg DM, THCA at 1732.26 mg/kg DM, THC at 157.49 mg/kg DM, and total THC at 1,676.68 mg/kg DM.

**Table 2:** Phytonutrient contents and antioxidant activities of the *Cannabis sativa* L. residue (CSR) powder

Item	Content
Chemical composition	
Dry matter (DM), g/kg	970
Organic matter, g/kg DM	763
Crude protein, g/kg DM	295
Ether extract, g/kg DM	61
Neutral detergent fiber, g/kg DM	627
Acid detergent fiber, g/kg DM	304
Gross energy, Kcal/kg DM	3575
Bioactive compounds^1^	
Total phenolic, mM GAE/g DM	4663.01
CBDA, mg/kg DM	1560.88
CBD, mg/kg DM	7829.28
CBN, mg/kg DM	1574.34
Total CBD, mg/kg DM	9198.17
THCA, mg/kg DM	1732.26
THC, mg/kg DM	157.49
Total THC, mg/kg DM	1,676.68
Antioxidant activities	
DPPH, IC50 (mg/mL)	12.78
ABTS, IC50 (mg/mL)	18.38
FRAP, mM Fe^2+^/g DM of sample	1664.49

^1^*GAE* gallic acid equivalent, *CBDA* cannabidiolic acid, *CBD* cannabidiol, *CBN* cannabinol, *THCA* tetrahydrocannabinolic acid, *THC* delta-9-tetrahydrocannabinol, Total CBD was calculated based upon CBD + 0.877 * CBDA (Stevens et al.) [53], Total THC was calculated based upon THC + 0.877 * THCA (Stevens et al.) [53]. IC_50_; the 50% half maximal inhibitory concentration (mg/mL), mM Fe^2+^/g DM pf sample; µM ferrous ion per DM pf sample. nd; not detected

### In vitro gas production kinetics and nutrient degradability

**Fig. 4:**
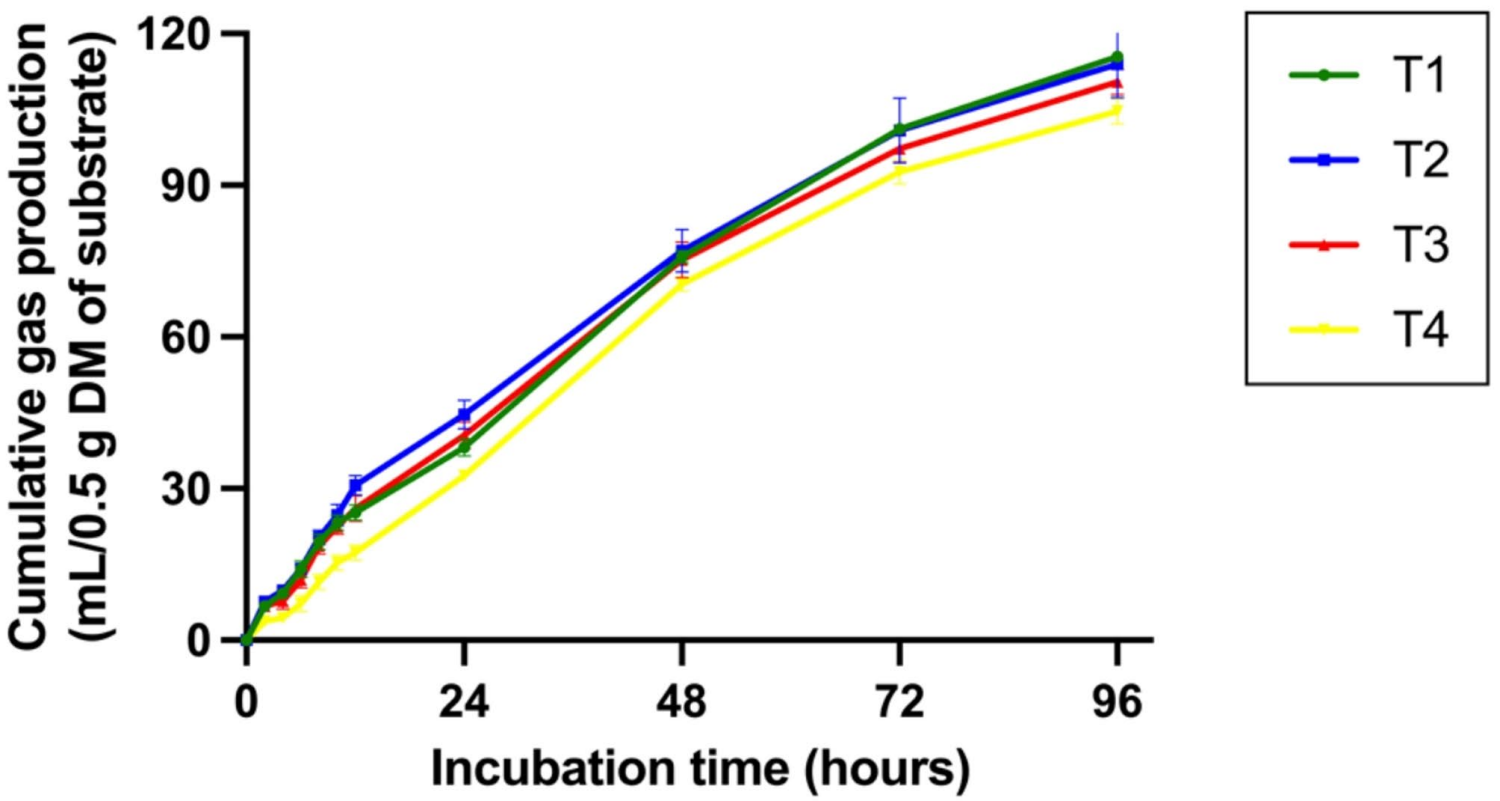
Effect of *Cannabis sativa* L. residue (CSR) powder supplementation levels on in vitro cumulative gas production (ml/0.5 g DM of substrate) after 0–96 h of incubation. The dietary treatments (T1-T4) were supplemented with CSR powder at concentrations of 0%, 0.5%, 1%, and 2% of the total DM substrate, respectively

The cumulative gas production (Table S2) is depicted in Fig. 4 as gas production curves, and Table 3 presents the results of gas kinetics production and in vitro nutrient digestibility. The gas production from the immediately soluble fraction (a) was significantly lowest (P < 0.05) in the group supplemented with 2% CSR of the total substrate (T4) compared to the other groups. The gas production from the insoluble fraction (b) and the potential extent of gas production (|a|+b) were significantly decreased (*P* < 0.05) in the CSR-supplemented groups compared to the control group. was significantly lowest (*P* < 0.05) in the group supplemented with 2% CSR of the total substrate (T4) compared to the other groups. Cumulative gas production at 96 h of incubation was significantly reduced (*P* < 0.05) in the group supplemented with 2% CSR of the total substrate (T4) compared to the control group, whereas the gas production rate constant for the insoluble fraction (c) did not differ among the treatment groups. Additionally, in vitro nutrient digestibility (IVDMD and IVOMD) at both 12 and 24 h was not affected by treatment or increased incubation time, as shown in Table 3.

**Table 3:** Effects of supplementation with *Cannabis sativa* L. residue (CSR) powder on gas production kinetics and nutrient digestibility after 12 and 24 h of in vitro incubation

Parameters	Treatment^1^				SEM	Contrast^2^		
	T1	T2	T3	T4		L	Q	C
Gas production kinetics^3^								
a, mL	2.16^a^	2.21^a^	1.01^a^	−2.24^b^	0.71	0.002	0.120	0.712
b, mL	171.40^a^	143.45^b^	147.10^b^	150.60^b^	5.05	0.064	0.020	0.577
c, mL/h	0.012	0.017	0.015	0.010	0.01	0.461	0.061	0.312
|a|+b, mL	173.56^a^	145.66^b^	148.11^b^	152.84^b^	5.12	0.618	0.017	0.657
Cumulative gas at 96 h, mL	115.45^a^	113.91^ab^	110.49^ab^	104.61^b^	1.86	0.039	0.461	0.963
IVDMD, g/kg								
12 h	37.82	38.43	37.55	37.32	0.37	0.582	0.660	0.615
24 h	48.95	50.40	48.27	48.47	0.44	0.856	0.530	0.542
IVOMD, g/kg DM								
12 h	43.18	44.41	43.40	43.22	0.43	0.396	0.497	0.188
24 h	55.89	58.24	55.79	56.13	0.52	0.712	0.358	0.154

^1^ Treatment: T1; 0% CSR, T2; 0.5% CSR, T3; 1.0% CSR, T4; 2.0% CSR. ^2^ Contrast: L; linear contrast, Q; quadratic contrast, C; cubic contrast. ^3^Gas production kinetics: a; the gas production from the immediately soluble fraction (mL), b; the gas production from the insoluble fraction (mL), c; the gas production rate constant for the insoluble fraction (mL/h), |a| + b; the potential extent of gas production (mL). ^a–c^ Means with different superscript letters show differences among treatments at each incubation time (*P* < 0.05). SEM; standard error of means

### Ruminal pH and ammonia-nitrogen (NH_3_-N) concentration

The in vitro pH and NH_3_-N concentrations at 12 h and 24 h of incubation are presented in Table 4. The pH values did not differ significantly among the treatment groups at either 12–24 h of incubation. However, NH_3_-N concentrations significantly increased in a quadratic pattern (*P* < 0.05) at 12 and 24 h of incubation with increasing levels of CSR supplementation. The T2, T3, and T4 groups exhibited significantly higher NH_3_-N concentrations than the T1 group at both time points (*P* < 0.05).

**Table 4:** Effects of the *Cannabis sativa* L. residue (CSR) powder supplementation level on the ruminal pH and ammonia-nitrogen (NH_3_-N) concentration after 12 and 24 h of in vitro incubation

Parameters	Treatment^1^				SEM	Contrast^2^		
	T1	T2	T3	T4		L	Q	C
Rumen pH								
12 h	6.39	6.35	6.34	6.38	0.01	0.138	0.236	0.126
24 h	6.86	6.84	6.83	6.84	0.01	0.227	0.115	0.554
NH_3_-N concentration, mg/dL								
12 h	26.58^c^	28.16^a^	27.98^b^	27.57^b^	0.27	0.165	0.049	0.341
24 h	32.54^b^	33.89^a^	33.95^a^	33.84^a^	0.26	0.123	0.041	0.255

^1^ Treatment: T1; 0% CSR, T2; 0.5% CSR, T3; 1.0% CSR, T4; 2.0% CSR. ^2^ Contrast: L; linear contrast, Q; quadratic contrast, C; cubic contrast. ^a–c^ Means with different superscript letters show differences among treatments at each incubation time (*P* < 0.05). SEM; standard error of means

### Volatile fatty acid (VFA) concentration and methane (CH_4_) production

**Fig. 5:**
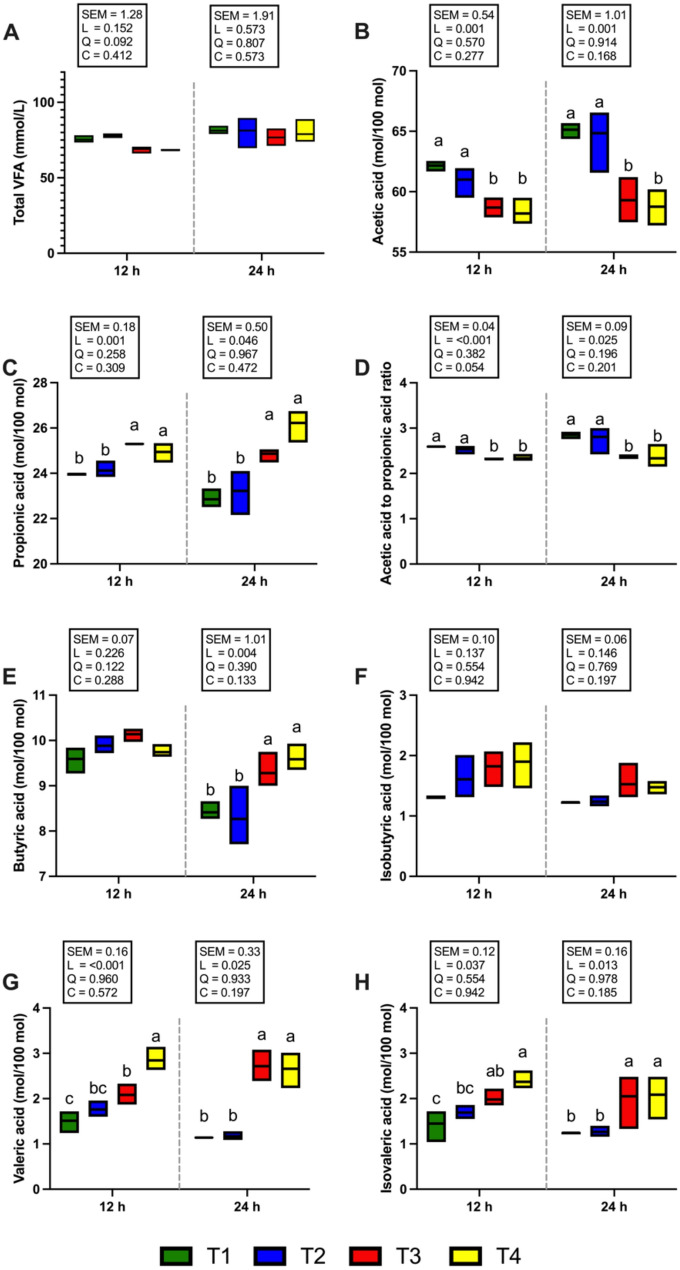
Effects of supplementation with *Cannabis sativa* L. residue (CSR) powder on volatile fatty acid (VFA) production after 12 and 24 h of in vitro incubation. **(A)** Total VFAs (mmol/L), **(B)** acetic acid (mol/100 mol), **(C)** propionic acid (mol/100 mol), **(D)** acetic acid to propionic acid ratio (mol/100 mol), **(E)** butyric acid (mol/100 mol), **(F)** isobutyric acid (mol/100 mol), **(G)** valeric acid (mol/100 mol), and **(H)** isovaleric acid (mol/100 mol). T1 (green bar); 0% CSR, T2 (blue bar); 0.5% CSR, T3 (red bar); 1% CSR, T4 (yellow bar); 2% CSR. ^a–c^ Means with different superscript letters represent differences among treatments at each incubation time (*P* < 0.05). SEM, standard error of means; L, linear contrast; Q, quadratic contrast; C, cubic contrast

**Fig. 6:**
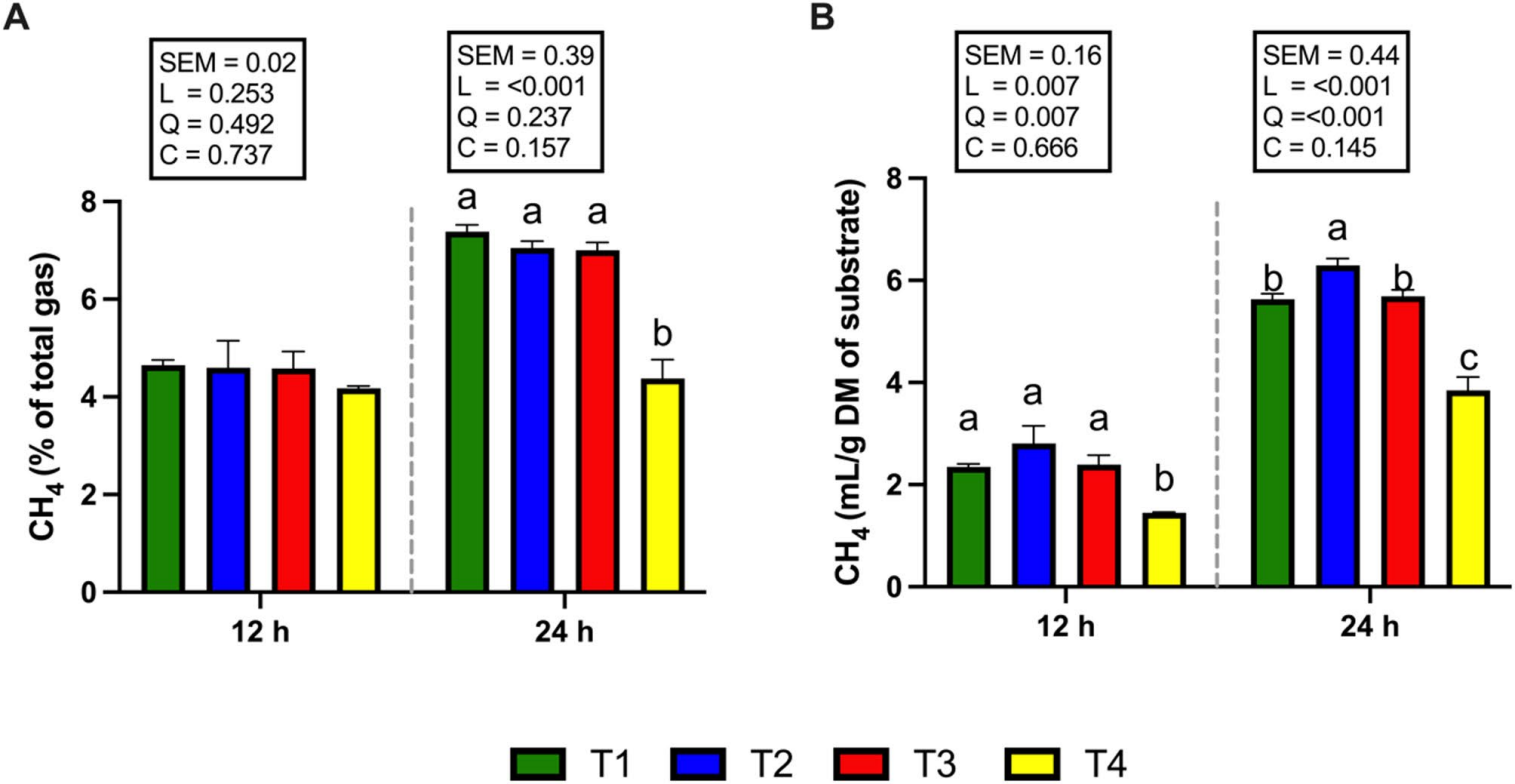
Effects of *Cannabis sativa* L. residue (CSR) powder supplementation on methane production (CH_4_) after 12 and 24 h of in vitro incubation. **(A)** CH_4_ (% of total gas, **(B)** CH_4_ (mL/g DM of substrate). T1 (green bar); 0% CSR, T2 (blue bar); 0.5% CSR, T3 (red bar); 1% CSR, T4 (yellow bar); 2% CSR. ^a–c^ Means with different superscript letters represent differences among treatments at each incubation time (*P* < 0.05). SEM, standard error of means; L, linear contrast; Q, quadratic contrast; C, cubic contrast

Figure 5 shows the total VFA and individual VFA proportions. The concentration of total VFAs did not differ among the treatments after 12 and 24 h of incubation (Fig. 5A). The T3 and T4 groups presented a significantly greater (*P* < 0.05) molar percentage of propionic acid at 12 and 24 h of incubation (Fig. 5A). On the other hand, when the supplementation level was increased to 1 and 2% of the total DM substrate (T3 and T4 groups), the proportion of acetic acid and the acetic acid to propionic acid ratio were significantly decreased (*P* < 0.05) at both 12 and 24 h of incubation (Fig. 5B, D). At 24 h of incubation, the molar proportion of butyric acid was significantly higher (*P* < 0.05) in the T3 and T4 groups compared with T1 and T2. However, no significant differences were observed among treatments at 12 h (Fig. 5E). None of the CSR treatments affected the molar percentage of isobutyric acid at 12 and 24 h of incubation (Fig. 5F). At both 12 and 24 h of incubation, the molar percentages of valeric and isovaleric acids were significantly higher (*P* < 0.05) in the T3 and T4 groups than in the T1 group, whereas the T2 group showed no significant difference (Fig. 5G, H). Interestingly, CH_4_ production after 12 and 24 h of incubation was significantly reduced (*P* < 0.05; Fig. 6B) in the 2% CSR supplementation group (T4), which also exhibited the lowest CH_4_ yield (mL/g DM of substrate) compared with the other treatment groups. Specifically, compared with the T1 group, the T4 group presented the most pronounced reduction in CH_4_ production at both 12 h (a reduction of 38.03%, *P* < 0.05) and 24 h (a reduction of 34.87%, *P* < 0.05) of incubation. Similarly, the proportion of CH_4_ to total gas production (%) in response to 2% CSR supplementation (T4) was significantly lower at 24 h of incubation (*P* < 0.05; Fig. 6A).

### Relative quantification of specific ruminal microbes and in situ degradation

The effects of CSR supplementation on the specific rumen microbial population are presented in Table 5. At the species level, the results of this study indicated that CSR supplementation had an effect on cellulolytic bacteria, specifically *R. flavefaciens*. The population of *R. flavefaciens* increased significantly (*P* < 0.05) in the T2 group compared with the T1, T3, and T4 groups after 12 and 24 h of incubation. However, compared with the T1 and T2 groups, CSR supplementation at 1 and 2% of total DM substrate (T3 and T4 groups) significantly decreased the population of *R. flavefaciens* after 24 h of incubation (*P* < 0.05). Similarly, the population of *Methanobacteriales* was significantly higher (*P* < 0.05) in the 0.5% CSR supplementation group (T2) than in the T1, T3, and T4 groups after 12 and 24 h of incubation. Furthermore, the quantity of *Methanobacteriales* significantly decreased with increasing CSR doses at 1 and 2% of total DM substrate (T3 and T4 groups) at both 12 and 24 h of incubation. The populations of *F. succinogenes*, *R. albus*, and *M. elsdenii* were not significantly different among the treatment groups after 12 and 24 h of incubation. For in situ degradation, Figure S1 and Table S2 present information about the percentage of DM and OM disappearance at different incubation times, along with the degradation kinetics and total disappearance observed at 96 h of incubation, which are listed in Table 6. CSR supplementation had no effect on the ruminal degradation kinetics, EDDM, or EDOM of the experimental diets.

**Table 5:** Effects of the *Cannabis sativa* L. residue (CSR) powder supplementation level on the rumen microbial population after 12 and 24 h of in vitro incubation

Relative quantification of selected rumen microbial population, % of total bacteria	Treatment^1^				SEM	Contrast^2^		
	T1	T2	T3	T4		L	Q	C
*F. succinogenes*, 10^_3^								
12 h	1.57	1.74	1.66	1.15	0.084	0.294	0.240	0.885
24 h	1.03	1.14	1.08	0.99	0.087	0.885	0.592	0.887
*R. albus*, 10^−3^								
12 h	1.64	1.66	1.09	0.96	0.147	0.058	0.798	0.411
24 h	1.53	1.60	1.24	1.21	0.095	0.138	0.794	0.370
*R. flavefaciens*, 10^−3^								
12 h	3.84^b^	5.60^a^	3.10^b^	3.17^b^	0.441	0.066	0.034	0.055
24 h	4.39^b^	5.67^a^	2.62^c^	2.85^c^	0.536	0.230	0.013	0.100
*M. elsdenii*, 10^−6^								
12 h	1.73	1.75	2.23	2.19	0.131	0.130	0.896	0.411
24 h	0.91	1.50	1.53	1.63	0.194	0.244	0.549	0.730
*Methanobacteriales*, 10^−3^								
12 h	2.09^b^	3.30^a^	1.25^c^	1.44^c^	0.341	0.146	0.005	0.147
24 h	1.69^b^	2.87^a^	1.23^c^	1.19^c^	0.286	0.125	0.004	0.122

^1^ Treatment: T1; 0% CSR, T2; 0.5% CSR, T3; 1.0% CSR, T4; 2.0% CSR. ^2^ Contrast: L; linear contrast, Q; quadratic contrast, C; cubic contrast. ^a–c^ Means with different superscript letters show differences among treatments at each incubation time (*P* < 0.05). SEM; standard error of means

**Table 6:** Effects of the *Cannabis sativa* L. residue (CSR) powder supplementation level on in situ degradation characteristics

Parameters	Treatment^1^				SEM	Contrast^2^		
	T1	T2	T3	T4		L	Q	C
DM degradability								
a, %	23.94	24.844	26.514	24.53	0.49	0.432	0.179	0.330
b, %	65.87	62.90	62.32	60.07	0.85	0.075	0.843	0.618
c, %/h	0.016	0.017	0.016	0.019	0.01	0.678	0.374	0.251
|a|+b, %	89.80	87.75	88.83	84.60	1.15	0.240	0.668	0.467
DM disappearance at 96 h, %	74.66	73.76	74.43	73.99	0.40	0.784	0.828	0.585
EDDM, %	40.15	40.51	41.85	40.99	0.72	0.100	0.210	0.154
OM degradability								
a, %	20.43	20.54	22.23	19.91	0.48	0.978	0.271	0.261
b, %	75.23	74.13	72.97	70.35	1.00	0.141	0.714	0.878
c, %/h	0.016	0.016	0.016	0.019	0.01	0.678	0.374	0.251
|a|+b, %	95.66	94.67	95.20	90.26	1.28	0.257	0.498	0.588
OM disappearance at 96 h, %	78.28	77.22	77.77	77.43	0.46	0.718	0.772	0.654
EODM, %	38.95	38.56	39.94	39.02	0.26	0.499	0.605	0.127

^1^ Treatment: T1; 0% CSR, T2; 0.5% CSR, T3; 1.0% CSR, T4; 2.0% CSR. ^2^ Contrast: L; linear contrast, Q; quadratic contrast, C; cubic contrast. EDDM; effective degradability of dry matter, EDOM; effective degradability of organic matter. SEM; standard error of means

## Discussion

Previous studies have shown that structural analogs of coenzyme M competitively and specifically inhibit MCR activity, resulting in a reduction in methane production [18, 61, 62]. The mechanism of action of these structural analogs of coenzyme M involves their ability to enter the active site of MCR, occupy the spatial position of coenzyme M, and block the biochemical reactions in which coenzyme M and coenzyme B combine to generate methane [63]. Consequently, when the entire active site and narrow channel were used as target sites for virtual screening, we found that CBD and THC could pass through the narrow channel and form hydrogen bonds with adjacent amino acid residues at the activation site on the basis of the results of the molecular docking approach. Our investigation revealed that the key residues (Met324, Met480, and Val482) of the MCR receptor play important roles in methanogenesis inhibition. This finding was consistent with previous research by Liu et al. [15], who reported that rosmarinic acid could reach the activation site through the narrow channel of MCR and form interactions, triggering narrow channels with Met324, Met480, Asn481, Asx367, and Val482, resulting in a reduction in methane emission. Moreover, the majority of the molecular interactions between the MCR receptor and the two compounds were preliminarily observed via hydrogen bonding (Tyr367 and Ala479) and hydrophobic interactions (Arg270, Phe361, Phe362, Tyr367, Gly368, Gly369, Ile380, Met480, and Val482) via molecular docking studies. All the key residues in the active site were predicted to inhibit MCR activity via phytochemicals [11]. Additionally, we utilized MD simulations to investigate various aspects of the ligand-protein complexes, such as their internal movement, conformational alterations, interaction mechanisms, and binding stability [64]. The computational results suggested that CBD and THC may be potent inhibitors that can specifically inhibit the activity of MCR, thereby reducing methane emissions. CBD and THC are natural phytonutrients that have been found in *C. sativa*, *C. indica*, and *M. speiosa* [65]. In addition to CBD and THC, researchers have identified bioactive secondary metabolites in the phytochemical categories of these plants, such as condensed tannins, saponins, TPC, THCA, and CBDA, which are accompanied by high antioxidant capacity in super plants [66]; however, their methane mitigation effects are not comparable to those of CBD and THC. As natural plant extracts, various studies have shown that CBD and THC exhibit antimicrobial, antiprotozoal, antimethanogenic, and antioxidant properties [65, 67]. To verify the methane inhibitory effect of this compound, we conducted an in vitro rumen fermentation experiment, as well as an in situ experiment, to study its impact on digestibility in the rumen. 

With respect to the phytonutrients of CSR, the contents of phytochemicals and their antioxidant activities differ significantly among individual medicinal plants. In this study, we demonstrated that CSR contains bioactive compounds such as phenolic compounds, CBDA, CBD, CBN, THCA, and THC. These bioactive compounds may contribute to the antioxidant activity of the CSR. The TPC and antioxidant activities of CSR partially align with findings from several other studies [65, 68, 69]. Sopian et al. [42] reported that the antioxidant activities of *C. sativa* residues, including leaves and stalks, were 236.94 mM TE/g (DPPH), 188.73 mM TE/g (ABTS), and 0.04 mM Fe^2+^/g (FRAP). Additionally, *C. sativa* residues were found to contain 754.3 mg/kg CBD and 1,005.9 mg/kg THC. The variations in phytochemical content observed in previous studies may be attributed to differences in the plant parts, chemovars, extraction methods, and solvents used [70, 71]. Moreover, oven-drying at high temperatures may lead to partial degradation of heat-sensitive bioactive compounds in cannabis, which could affect both the concentration of these compounds and their methane-reducing efficacy. Therefore, caution should be exercised when applying such methods. However, in our experiment, the drying process was conducted at 60 °C for 48 h prior to testing. This temperature and duration did not appear to negatively impact the concentration of bioactive compounds. This is supported by previous research indicating that cannabinols are stable at 60 °C for up to 25 days [72].

In the present study, CSR supplementation impacted the immediately soluble fraction (a), insoluble fraction (b), potential extent of gas production (|a|+b), and cumulative gas content after 96 h of incubation. The current experimental hypothesis was that the potential of phytonutrients, including the TPC, CBD, and THC, along with the antioxidant capacity within the CSR could mitigate gas production resulting from ruminal microbial activity. High doses of different plant extracts have been shown to reduce the activity of rumen microbes and total gas production [73]. Although CSR supplementation reduced total gas production, increasing its inclusion level did not adversely affect other ruminal fermentation parameters, including IVDMD, IVOMD, rumen pH, and total VFA concentration. This is similar to the concept of the mode of action of phenolic acids involving their diffusion in microbial membranes, which leads to cytoplasmic acidification and subsequent cell death. This process affects bacterial membranes and contributes to the antimicrobial activity of phenolic acids [74, 75]. Similarly, Blaskovich et al. [67] reported that CBD and other cannabinoids have selective activity against a subset of gram-negative bacteria. These findings are in accordance with a previous study that demonstrated that gas production kinetics, including fraction a, fraction b, the potential extent of gas production (a + b), and cumulative gas at 96 h, was reduced in a diet supplemented with microencapsulated hemp (*Cannabis sativa L.*) leaf (which contains phytonutrient components such as TPC, CBD, and THC) at varying levels ranging from 4 to 8% of the total DM substrate [27, 76].

In this study, the pH value was not influenced by the treatments. The data for rumen fermentation and the activity of the microbial population fell within the normal range of pH values (6.34 to −6.86). It has been reported that ruminal microbial activity is negatively impacted when the pH falls below 5.0-5.5 [77]. However, the results of our study indicate that the pH of the tested CSR did not negatively influence fiber degradation. The pH in this study was in accordance with that in other studies by Van Soest [78] and Phupaboon et al. [79], who reported an optimal ruminal pH of 6.2–7.2 for microbial activity, especially for cellulolytic and/or proteolytic bacteria, to degrade fiber and protein to increase the carbon and nitrogen sources for microbiota growth in in vitrofermentation studies. In this study, the NH₃–N concentration at 12 and 24 h postincubation increased with increasing CSR supplementation. This could be due to the high crude protein content in CSR (295 g/kg DM). Additionally, the enhancement of the proteolysis process is likely influenced by the bioactive compounds present in the plant extract [80]. Ahmed et al. [81] reported that supplementation with plant-derived phytonutrients has the potential to increase ruminal NH_3_-N concentrations.

Ruminants depend on a symbiotic relationship with a diverse community of microbes in the anaerobic environment of the rumen to facilitate the breakdown of complex nutrients such as carbohydrates, proteins, and some organic polymers into their constituent monomers. The monomers undergo fermentation, resulting in the production of end products, including VFAs (the main end products of the ruminal fermentation of organic matter), free ammonia (NH_3_), carbon dioxide (CO_2_), and hydrogen (H_2_). Additionally, CO_2_ and H_2_ are transformed into CH_4_ by the activity of methanogens, including *Methanopyrales*, *Methanocellales*, and *Methanomicrobiales* [82].

In the present study, the production of total VFAs was not affected by CSR supplementation. VFAs generated in the rumen through microbial fermentation serve as the primary energy source for ruminants [78]. The absence of any effect of CSR on the total VFA concentration in this study indicates that the dosage used did not adversely affect the nutrition of ruminants. Generally, the major VFAs that enter the bloodstream and provide energy and a substrate for anabolic processes in ruminants are acetic acid, propionic acid and butyric acid [83]. Acetic acid and butyric acid can promote CH_4_ production, whereas propionic acid formation can be considered a competitive pathway for H_2_ sink in the rumen fermentation [84, 85]. In the term of mechanism, the producers of CO_2_ and H_2_ are organisms that produce acetatic acid [C_6_H_12_O_6_ + 2H_2_O —>2C_2_H_4_O_2_ (acetic acid) + 2CO_2_ + 8 H] and butyric acid [C_6_H_12_O_6_ + C_4_H_8_O_2_ (butyric acid) —>2CO_2_ + 4 H] in the fermentation pathway, while the propionic acid fermentation pathway [C_6_H_12_O_6_ + 4 H —>2C_3_H_6_O_2_ (propionic acid) + 2H_2_O] does not liberate CO_2_ or H_2_ [83]. Therefore, the observed shift in the fermentation profile, particularly the increased proportion of propionic acid acting as a hydrogen sink, represents one of the strategies that can mitigate methane emissions. Notably, CSR supplementation (at 1 and 2% total DM substrate) resulted in a greater proportion of propionic acid but a lower proportion of acetic acid, acetic acid to propionic acid ratio, and CH_4_ production. The influence of plant secondary compounds may contribute to the production of propionic acid in environments with excess hydrogen [86]. Under these conditions, hydrogen can be utilized for propionate synthesis instead of being the primary substrate for methane production [87]. Because methanogenesis is one of the main H_2_ sinks in rumen fermentation, the increase in H_2_ emission can be directly associated with the inhibition of methanogenesis [20]. Our hypothesis in this study was that CBD and THC can block the biochemical reaction in which coenzyme M and coenzyme B combine to generate methane at the active site of MCR, leading to an increase in H_2_ emissions in the rumen. Previous studies on methane inhibitors by 3-NOP (a specific inhibitor of MCR) reported a significant increase in hydrogen emissions under methane inhibition, whereas carbon dioxide remained unchanged [20]. Hydrogen (H₂) in the rumen exists in two forms: gaseous and dissolved in the rumen liquor [85]. In vitro studies have shown that the accumulation of H₂ can lead to the production of fermentation end products, such as propionate and butyrate, which act as net sinks for H₂. The dissolved H_2_ concentration was positively correlated with the molar proportions of butyric acid (R^2^ = 0.621) and propionic acid (R^2^ = 0.904) and negatively correlated with the ratio of acetic acid to propionic acid (R^2^ = 0.853) and the molar proportion of acetic acid (R^2^ = 0.902) [88]. Similarly, Melgar et al. [89] reported that other potential sinks for H_2_ include some rumen VFAs (specifically propionic acid but also butyric acid and valeric acid), formate, ethanol, and above all, the dissolved H_2_ pool in the rumen. In particular, the propionic acid concentration generally increases when rumen methanogenesis is inhibited [20, 89], which was also observed in the present study.

The results of the present study indicate that CBD and THC in CSR may be potent inhibitors that can specifically inhibit the activity of MCR, thereby reducing methane emissions. Total phenolic compounds are known for their antimicrobial properties and their potential to modulate ruminal fermentation processes, which can mitigate methane emissions [3], and previous in vitro studies that assessed the methane mitigation ability and mode of action of phenolic compounds in plants revealed that they decrease CH_4_ production in the presence of antimethanogenic compounds [74]. Furthermore, these findings are consistent with the reduction in the populations of *R. flavefaciens* and *Methanobacteriales* in this study in terms of phytonutrients, including the TPC. This process affects bacterial membranes and contributes to the antimicrobial activity of phenolic acids [74, 75]. This may be due to the susceptibility of plants to bioactive compounds, particularly phenolic compounds, which are abundant in CSR. This observation is consistent with that of Kim et al. [90], who reported that the inclusion of a flavonoid-rich plant extract in the diet caused a reduction in the population of *R. flavefaciens*. Similarly, several earlier studies [91–93] have shown that plant secondary compounds such as flavonoids, phlorotannins, and condensed tannins lead to a decrease in the population of *R. flavefaciens*, and Phupaboon et al. [76] reported that increasing the concentration of mHLE supplemented with TPC compounds and antioxidant capacity consistently reduced the population of methanogens and protozoa, especially *Methanobacteriales* (e.g., *Methanobacterium formicicum*) and *Archaeoglobus fulgidus*. Matra et al. [80] highlighted that incorporating microencapsulated bioactive compounds, especially phenolic and flavonoid compounds derived from *Mitragyna* leaf extracts, may contribute to a decline in the *Methanobacteriales* population. In this study, the T2 group exhibited a higher abundance of *R. flavefaciens* and *Methanobacteriales* compared to the control group, while their populations decreased at the higher supplementation levels in the T3 and T4 groups. These results may be attributed to the effects of bioactive compounds present in CSR, as phytonutrient supplementation at an appropriate level has been shown to enhance microbial activity, particularly in cellulolytic bacterial groups (e.g., *F. succinogenes*,* R. albus* and *R. flavefaciens*) [3]. Bioactive compounds activity alters protein translocation, phosphorylation processes, ion gradients, electron transport, and other enzyme-dependent processes, which results in the impacted cellulolytic bacteria losing chemiosmotic control [94]. Therefore, bioactive compounds could be supplemented at suitable levels to support microbial activity, especially that of cellulolytic bacteria. *F. succinogenes*,* R. albus*, and *R. flavefaciens* have been identified as the main cellulolytic bacterial species in the rumen and increasing their abundance can improve fiber degradation in ruminants [95]. Previous studies by Matra et al. [80] reported that microencapsulated *Mitragyna* leaf extract supplementation, rich in bioactive compounds, at 6% of the total substrate led to a greater population of *R. flavefaciens* compared to the 4% and 8% inclusion levels in an in vitro study. Similarly, Chanjula et al. [96] found that dietary inclusion of dried *Mitragyna* (kratom) leaves, a rich source of plant secondary metabolites, at a dose of 4.44 g/goat increased *R. flavefaciens* abundance, while higher doses (5.55 and 6.66 g/goat) resulted in a population decline in an in vivo study. The observed increase in the *Methanobacteriales* population in the T2 group was consistent with the elevated abundance of *R. flavefaciens*. This association may be explained by the mutualistic relationship between fermentative ruminal bacteria and methanogens, in which the latter utilize CO₂ and H₂ produced by the former as substrates. Such interspecies hydrogen (H₂) transfer has been demonstrated in co-cultures of methanogens with *R. albus* and *R. flavefaciens*, supporting the notion that shifts in cellulolytic bacterial populations (*R. flavefaciens*) can influence methanogen (*Methanobacteriales*) abundance [62]. However, our results show that CBD and THC may be able to effectively inhibit the activity of MCR because supplementation with 1% and 2% CSR did not result in significant differences in the proportions of acetic acid and propionic acid, the acetic acid to propionic acid ratio, and the populations of *R. flavefaciens* and *Methanobacteriales*, whereas supplementation with 2% CSR resulted in significantly lower CH_4_ production. These results strengthen the validity of our hypothesis regarding the potential of CBD and THC in inhibiting the activity of MCR, beyond the effects observed from the plant secondary compounds in CSR. To our knowledge, little information is available regarding the effects of CSR on the in situ degradation of feedstuff. In the present study, the in situ degradability of DM and OM in the dietary treatments was not affected by CSR supplementation. Nonetheless, the observed effects in this study may not be solely attributed to the cannabinoids CBD and THC present in the CSR, but may also have been influenced by other bioactive constituents within its complex matrix, such as terpenes, flavonoids, or other secondary metabolites. These compounds may exert individual effects or act synergistically to influence rumen fermentation and methane production. Future research should focus on isolating and characterizing individual compounds or groups of compounds in CSR and evaluating their specific roles in modulating rumen microbiota and mitigating methane emissions. This will provide a clearer understanding of the underlying mechanisms involved. The aforementioned findings and discussion support the conclusion that incorporating CBD and THC as plant-derived feed additives holds substantial promise in practical applications. Notably, the positive outcome observed in vitro does not inherently imply commensurate effectiveness of the same treatment and dose in vivo. Therefore, animal experiments need to be conducted to verify the efficacy of CBD and THC as methane mitigation compounds and their effects on animal production, physiology, and health.

## Conclusion

According to computational assays, CBD and THC, the primary active compounds in CSR, were found to traverse a narrow channel and bind to the active sites of MCR. The resulting MCR–CBD and MCR–THC complexes exhibited high stability, suggesting that these compounds may have the potential to reduce ruminal methane production by inhibiting MCR activity. Moreover, in vitro and in situ assays revealed that supplementing CSR at 2% of dietary DM via a TMR diet reduced in vitro rumen fermentation enteric CH_4_ emissions by approximately 34%, without adversely affecting total VFA concentrations, in vitro nutrient digestibility, or in situ degradability. Furthermore, CSR supplementation altered the rumen methanogen community by decreasing the relative abundance of *Methanobacteriales* and increasing the proportion of propionic acid. Nevertheless, further investigations are warranted to elucidate the precise molecular mechanisms underlying the inhibitory effects of CBD and THC on MCR activity. In addition, comprehensive in vivo studies are essential to validate these findings and fully assess their implications for methane mitigation and ruminant health.

## Supplementary Information

Supplementary Material 1.

## Data Availability

Data is provided within the manuscript or supplementary information files.

## Abbreviations

ABTS: 2,2-azino-bis(3-ethylbenzthiazoline-6-sulphonic acid)
ADF: Acid detergent fiber
BES: 2-bromoethanesulfonate
BPS: 3-bromopropanesulfonate
BW: Body weight
CADD: Computer-aided drug discovery
CBD: Cannabidiol
CBDA: Cannabidiolic acid
CBN: Cannabinol
CES: 2-chloroethanesulfonate
CH_4_: Methane
Cl⁻: Chloride
CO₂: Carbon dioxide
CRD: Completed randomized design
CSR: Cannabis sativa L. residue
DM: Dry matter
DPPH: 2,2-diphenyl-1-picrylhydrazyl
ED: Effective degradability
EDDM: Effective degradability of dry matter
EDOM: Effective degradability of organic matter
FRAP: Ferric reducing ability power
GC: Gas chromatography
H_2_: Hydrogen
HMAs: Halogenated methane analogs
IVDMD: In vitro dry matter degradability
IVOMD: In vitro organic matter degradability
MCR: Methyl-coenzyme M reductase
MD: Molecular dynamics
Na⁺: Sodium
NDF: Neutral detergent fiber
NH_3_-N: Ammonia-nitrogen
OM: Organic matter
RMSD: Root-mean-square deviation
RMSF: Root-mean-square fluctuation
RT-PCR: Real-time polymerase chain reaction
SEM: Standard error of the mean
TCD: Thermal conductivity detector
THC: Delta-9-tetrahydrocannabinol
THCA: Tetrahydrocannabinolic acid
TMR: Total mixed ration
TPC: Total phenolic content
VFA: Volatile fatty acid
