# Supplementary material for: Harnessing bioactive compounds from Cannabis sativa residue to improve rumen fermentation and reduce methane production: in silico, in vitro, and in situ nylon bag studies
Source: BMC Vet Res. 2025 Oct 14;21:611. doi: 10.1186/s12917-025-04985-5 (PMC12522255; doi:10.1186/s12917-025-04985-5)
Supplement: Supplementary file 1 — Supplementary Material 1. [file 12917_2025_4985_MOESM1_ESM.docx]

**Supplementary material**

**Supplementary table**

**Table S1** qPCR primers

| **Target** | **Primer sequences (5'-3')** | **References** |
| --- | --- | --- |
| Total bacteria | CGGCAACGAGCGCAACCC | Denman and McSweeney, (2006) |
|  | CCATTGTAGCACGTGTGTAGCC |  |
| *Methanobacteriales* | GGGCTTGCTTTGGAAACTGTT | Yu et al., (2005) |
|  | CCCACCGATGTTCCTCCTAA |  |
| *Fibrobacter succinogenes* | GGTATGGGATGAGCTTGC | Koike and Kobayashi, (2001) |
|  | GCCTGCCCCTGAACTATC |  |
| *Ruminococcus albus* | CCCTAAAAGCAGTCTTAGTTCG | Denman et al., (2007) |
|  | CCTCCTTGCGGTTAGAACA |  |
| *Ruminococcus flavefaciens* | TCTGGAAACGGATGGTA | Yu et al., (2005) |
|  | CCTTTAAGACAGGAGTTTACA A |  |
| *Megasphaera elsdenii* | GACCGAAACTGCGATGCTAGA | Ouwerkerk et al., (2002) |
|  | TCCAGAAAGCCGCTTTCGCCACT |  |

**Table S2** *In vitro* cumulative gas production of diet supplemented with different dose levels of *Cannabis sativa* L. residue (CSR) powder at various incubation times.

| **Parameters** | **Treatment^1^** | | | | **SEM** | **Contrast^2^** | | |
| --- | --- | --- | --- | --- | --- | --- | --- | --- |
|  | **T1** | **T2** | **T3** | **T4** |  | **L** | **Q** | **C** |
| ***In vitro* cumulative gas production, ml/0.5 g DM** | | | | | | | | |
| GP2h | 6.75^a^ | 7.68^a^ | 6.72^a^ | 3.77^b^ | 0.57 | 0.006 | 0.009 | 0.959 |
| GP4h | 9.21^a^ | 9.81^a^ | 7.70^a^ | 4.46^b^ | 0.83 | 0.007 | 0.055 | 0.647 |
| GP6h | 14.05^a^ | 14.16^a^ | 11.97^a^ | 7.25^b^ | 1.12 | 0.007 | 0.074 | 0.962 |
| GP8h | 19.49^a^ | 20.33^a^ | 18.63^a^ | 11.54^b^ | 1.38 | 0.006 | 0.021 | 0.585 |
| GP10h | 23.24^a^ | 24.79^a^ | 22.40^a^ | 15.32^b^ | 1.45 | 0.008 | 0.023 | 0.896 |
| GP12h | 25.24^a^ | 30.60^a^ | 26.09^a^ | 17.32^b^ | 1.87 | 0.009 | 0.006 | 0.406 |
| GP24h | 38.17^cb^ | 44.68^a^ | 40.58^ab^ | 32.58^c^ | 1.76 | 0.041 | 0.010 | 0.393 |
| GP48h | 75.82 | 77.02 | 75.29 | 70.49 | 1.22 | 0.128 | 0.220 | 0.988 |
| GP72h | 101.19 | 100.84 | 97.27 | 92.64 | 1.63 | 0.065 | 0.456 | 0.861 |
| GP96h | 115.45^a^ | 113.91^ab^ | 110.49^ab^ | 104.61^b^ | 1.86 | 0.039 | 0.461 | 0.963 |

^1^ Treatment: T1; without CSR, T2; 0.5% CSR, T3; 1.0% CSR, T4; 2.0% CSR. ^2^ Contrast: L; linear contrast, Q; quadratic contrast, C; cubic contrast. ^a–c^ Means with different superscript letters show differences among treatments at each incubation time (P < 0.05). SEM; standard error of means.

**Table S3** *In situ* DM and OM disappearance of diet supplemented with different dose levels of *Cannabis sativa* L. residue (CSR) powder in the rumen at various incubation times.

| **Parameters** | **Treatment^1^** | | | | **SEM** | **Contrast^2^** | | |
| --- | --- | --- | --- | --- | --- | --- | --- | --- |
|  | **T1** | **T2** | **T3** | **T4** |  | **L** | **Q** | **C** |
| ***In Situ* DM disappearance , %** | | | | | | | | |
| 3 h | 25.91 | 27.38 | 28.14 | 26.83 | 0.69 | 0.257 | 0.062 | 0.153 |
| 6 h | 32.18 | 30.96 | 31.66 | 31.60 | 0.39 | 0.819 | 0.576 | 0.559 |
| 12 h | 35.60 | 37.57 | 37.80 | 37.57 | 0.43 | 0.116 | 0.185 | 0.699 |
| 24 h | 43.98 | 44.41 | 46.81 | 46.54 | 0.58 | 0.067 | 0.718 | 0.318 |
| 48 h | 59.06 | 58.18 | 58.85 | 58.36 | 0.42 | 0.774 | 0.860 | 0.596 |
| 72 h | 71.94 | 71.02 | 71.71 | 71.25 | 0.41 | 0.783 | 0.834 | 0.587 |
| 96 h | 74.66 | 73.76 | 74.43 | 73.99 | 0.40 | 0.784 | 0.828 | 0.585 |
| ***In Situ* OM disappearance, %** | | | | | | | | |
| 3 h | 22.75 | 23.79 | 24.39 | 22.92 | 0.65 | 0.508 | 0.076 | 0.146 |
| 6 h | 29.68 | 27.46 | 28.53 | 27.97 | 0.51 | 0.442 | 0.483 | 0.362 |
| 12 h | 33.95 | 34.88 | 34.89 | 34.66 | 0.94 | 0.586 | 0.509 | 0.856 |
| 24 h | 43.07 | 42.59 | 45.33 | 45.34 | 0.61 | 0.104 | 0.822 | 0.263 |
| 48 h | 60.62 | 59.37 | 59.91 | 59.47 | 0.49 | 0.618 | 0.752 | 0.637 |
| 72 h | 75.32 | 74.22 | 74.78 | 74.41 | 0.12 | 0.703 | 0.770 | 0.652 |
| 96 h | 78.28 | 77.22 | 77.77 | 77.43 | 0.46 | 0.718 | 0.772 | 0.654 |

^1^ Treatment: T1; without CSR, T2; 0.5% CSR, T3; 1.0% CSR, T4; 2.0% CSR. ^2^ Contrast: L; linear contrast, Q; quadratic contrast, C; cubic contrast. SEM; standard error of means.


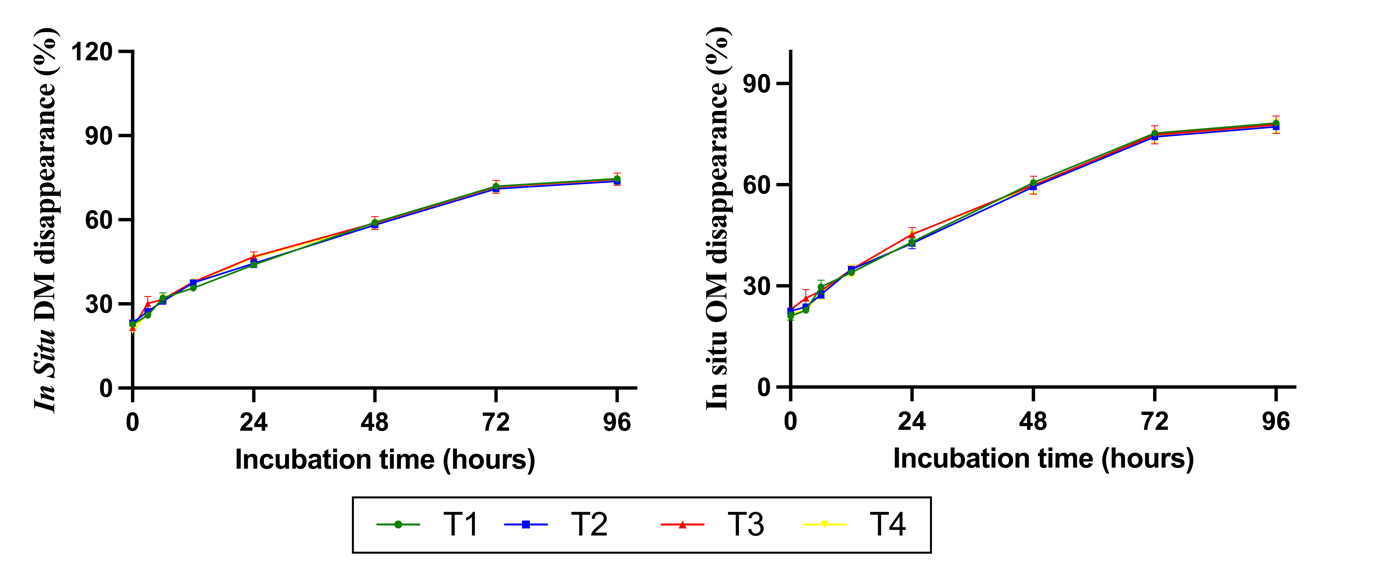


**Figure 1.** Effect of the *Cannabis sativa* L. residue (CSR) powder supplementation level on *in situ* **(A)** DM disappearance (%) and **(B)** OM disappearance (%) after 0–96 h of incubation in the rumen. The dietary treatments (T1-T4) were supplemented with CSR powder at concentrations of 0%, 0.5%, 1%, and 2% of the total DM substrate, respectively.

**Reference**

Denman SE, McSweeney CS. Development of a real-time PCR assay for monitoring anaerobic fungal and cellulolytic bacterial populations within the rumen. FEMS Microbiol Ecol. 200; 658:572-582.

Denman SE, Tomkins NW, McSweeney CS. Quantitation and diversity analysis of ruminal methanogenic populations in response to the antimethanogenic compound bromochloromethane. FEMS Microbiol Ecol. 2007; 62: 313-322.

Koike S, Kobayashi Y. Development and use of competitive PCR assays for the rumen cellulolytic bacteria: *Fibrobacter succinogenes*, *Ruminococcus albus* and *Ruminococcus flavefaciens*. FEMS Microbiol. 2001; 204: 361-366.

Ouwerkerk D, Klieve AV. Enumeration of *Megasphaera elsdenii* in rumen contents by real‐time Taq nuclease assay. J Appl Microbiol. 2002; 92: 753-758.

Yu Y, Lee C, Kim J, Hwang S. Group-specific primer and probe sets to detect methanogenic communities using quantitative real-time polymerase chain reaction. Biotechnol Bioeng. 200; .89: 670-679.
